# Supplementary material for: Genetic comparisons of interleukin-17 reveal a framework for complex signaling evolution
Source: bioRxiv. 2026 Apr 14:2026.04.13.718218. Preprint. [Version 1] doi: 10.64898/2026.04.13.718218 (PMC13105070; doi:10.64898/2026.04.13.718218)
Supplement: Supplement 3 [file media-3.zip › Supplemental files for paper/ERC/IL17xIL17_comparisons/IL17C_IL25.pdf]

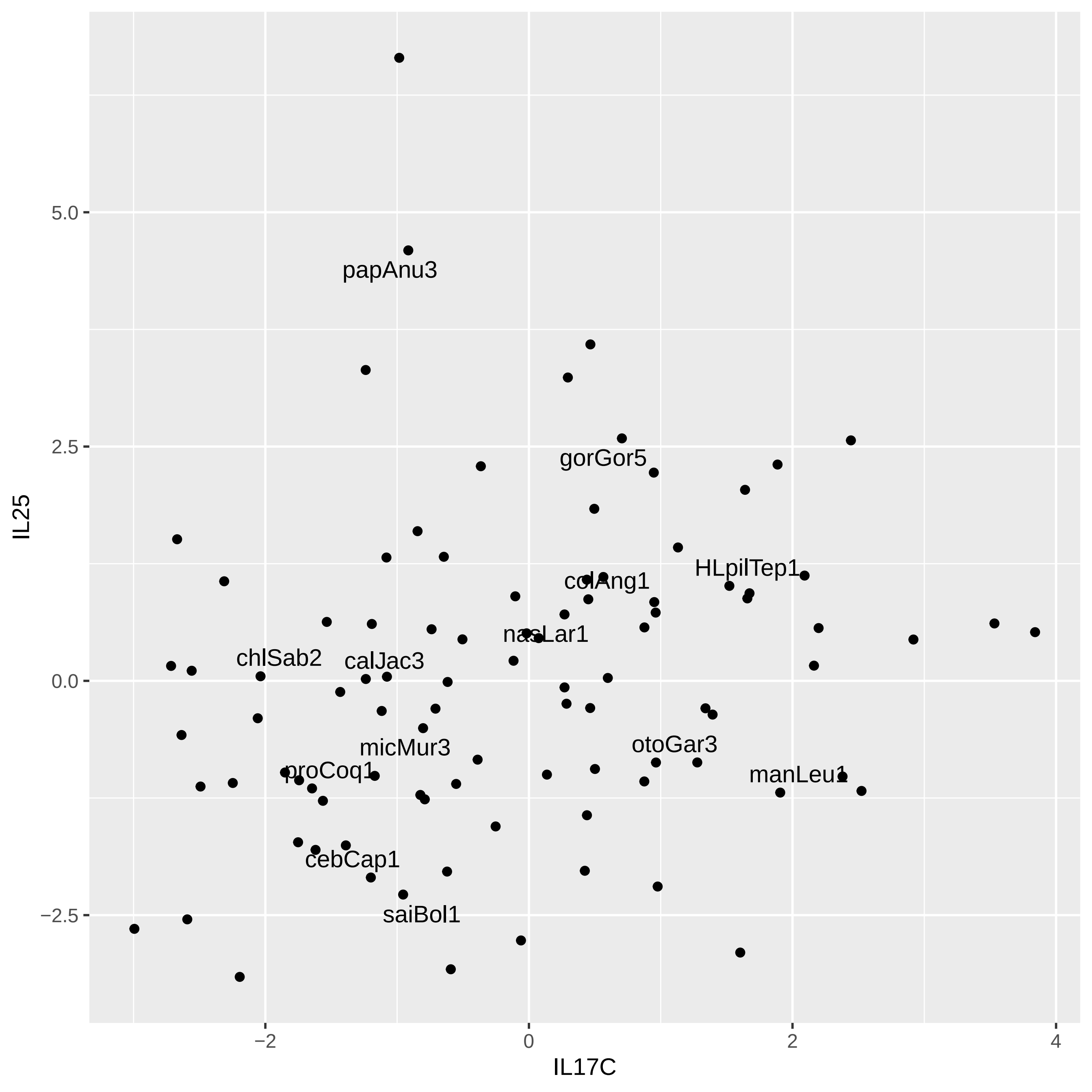

papAnu3
gorGor5
HLpilTep1
colAng1
nasLar1
chlSab2
calJac3
micMur3
otoGar3
proCoq1
manLeu1
cebCap1
saiBol1
5.0
2.5
0.0
−2.5
−2
0
2
4
IL25
IL17C
